# Temperature effect on seawater leaching of Pb, Zn, and F from waste rock at Ivittuut cryolite mine in South Greenland

**DOI:** 10.1007/s11356-026-37562-w

**Published:** 2026-03-11

**Authors:** Ninni E. O. Jeremiassen, Yu Jia, Violeta Hansen, Henrik Friis, Morten Birch Larsen, Maia Olsen, Thomas Ulrich

**Affiliations:** 1 Department of Geoscience, University of Aarhus, Aarhus, Denmark; 2 Department of Environment and Mineral Resources, Greenland, Institute for Natural Resources, Nuuk, Greenland; 3 Department of Radiation Physics, University of Gothenburg, Gothenburg, Sweden; 4 Natural History Museum, University of Oslo, Oslo, Norway; 5 Rambøll, Section of Contaminated Soil and Groundwater, Aarhus, Denmark; 6 Institute of Geotechnology and Mineral Resources, Department for Geosciences, Clausthal University of Technology, Clausthal-Zellerfeld, Germany

**Keywords:** Arctic, Pollution, Mineral weathering, Humidity cell test, Mine waste, Seawater leaching

## Abstract

Pollution from mining waste poses environmental risks and can adversely impact nearby communities. Understanding the weathering processes of mining waste and pollutant release is crucial for effective environmental remediation. At Ivittuut, South Greenland, cryolite was mined for 130 years. Waste rock (WR) from the mine was disposed of along the coastline of Arsuk Fjord. The WR contains sulfides such as pyrite (FeS_2_), galena (PbS), sphalerite (ZnS), and chalcopyrite (CuFeS_2_), with minor arsenopyrite (FeAsS) and molybdenite (MoS_2_). Heavy metals, mainly Pb and Zn, leach from WR into the fjord. Monitoring over 40 years estimates the annual release of dissolved Pb into Arsuk Fjord to be 400–1000 kg. We performed a 40-week leaching experiment, using WR from Ivittuut and seawater in a Humidity Cell Test (HCT). The experiment was conducted at room temperature and 2 °C. Results show that at room temperature the concentration of Pb and Zn leached from the WR is tenfold higher than at 2 °C. Pb and Zn concentrations in leachates exceeded Greenland Water Quality Criteria (GWQC) by a factor of 20. Fluorine (F) concentrations were 10–15 times higher than GWQC. Compared to room temperature, 2 °C delayed and reduced leaching rates of Pb, Zn, and F indicate that contaminant leaching from WR in Ivittuut is slower under colder conditions, implying that Arctic climatic conditions slow down the metal mobilization, reducing Pb and Zn release at Ivittuut during winter months.

**Supplementary Information:**

The online version contains supplementary material available at 10.1007/s11356-026-37562-w.

## Introduction

Arctic environmental contamination arises from both local industrial activities and long-range transport of contaminants via oceanic currents and atmospheric circulation (Law et al. 2017; Sonne et al. 2021). These pollutants can bioaccumulate, posing risks to Arctic biota and local communities reliant on traditional diets (Hembrom et al. 2020). The lack or improper management of mining waste can result in environmental pollution due to the release of toxic elements through waste weathering. Drilling, blasting, and ore grinding create fresh mineral surfaces, whilst exposure to air and water results in weathering and mobilization of pollutants from mine waste into the environment (Jamieson et al. 2015; Park et al. 2014).

Improper disposal of mine waste, especially in historical times when waste management was less regulated, can lead to acid rock drainage (ARD) (Søndergaard and Mosbech 2022). This is particularly problematic for sulfide-rich materials, as ARD lowers pH and increases the mobility of toxic metals, severely impacting ecosystems (Ek and Renberg 2001; Jia et al. 2023; Salonen et al. 2006). However, pollutant release into the environment from the mine waste also occurs under neutral or alkaline conditions through natural weathering (Lindsay et al. 2015). Effective waste management depends on the mineral composition of the ore and waste and site weather and climate.

Temperature impacts the leaching rate of contaminants from waste rock. In Arctic and subarctic climates, the lower temperatures reduce the rate of chemical weathering and the release of, e.g., heavy metals from mining waste, as lower temperatures slow down the oxidation of sulfide minerals and the dissolution of metals like Pb, Zn, and Cu (Ethier et al. 2012; Fu et al. 2023; Lu et al. 2021). Studies have shown that concentrations of metals in leachates from waste leaching experiments in Arctic environments are generally lower than at mid-latitude regions due to the slow rate of weathering processes at temperatures close to freezing (Fu et al. 2023; Lu et al. 2021).

In Greenland, there are some polluted legacy mining sites. These include pollutions observed from the Pb–Zn-sulfide mine at Maarmorilik (also known as Black Angel), the Pb-sulfide mine in Mestersvig (also known as Blyklippen), and the cryolite mine at Ivittuut (Bach et al. 2014; Søndergaard and Mosbech 2022). The mines were closed before environmental regulations were introduced in Greenland, and therefore, no environmental impact assessment was developed before mining commenced, no monitoring was performed during the active mining period, and no waste management was in place (Johansen et al. 1995; Søndergaard and Mosbech 2022). The contamination at the Greenland legacy sites is primarily sourced from the tailings and waste rock. These waste materials are still releasing Pb and Zn into the environment. Lead and zinc are non-degradable heavy metals that are toxic and hazardous to both biota and human health, with lead being classified as highly toxic (Collin et al. 2022; Sankhla et al. 2019).

The present study focuses on Pb and Zn environmental pollution at the legacy mine site at Ivittuut. Environmental monitoring at Ivittuut began in 1982, because of concerns about pollution in the Arsuk Fjord, and this led to the discovery of high Pb and Zn concentrations in comparison with Greenland Water Quality Criteria (GWQC) in water, sediment, and biota in the Arsuk Fjord (Johansen et al. 1995, 2010; Larsen and Geertz-Hansen 2016; MRA 2015). Fish in the Fjord are unaffected by the high concentrations of Pb and Zn in the water, while it is not recommended that mussels are consumed due to high concentrations of these elements in these samples (Johansen et al. 1995). The estimated Pb release from the site is approx. 400–1000 kg per year (Johansen et al. 2010). However, the long-term monitoring indicated that the release of Pb (and to a lesser degree Zn) is decreasing over time (Johansen et al. 1995, 2010). No other elements have been included in the monitoring program. The ore of the former mine was cryolite, a sodium-aluminum-fluoride mineral (Na_3_AlF_6_), but only the high-grade cryolite was shipped from the site. A substantial amount of low-grade cryolite rock was disposed of along the coast of Arsuk Fjord. Limited investigations have been done on the alteration of cryolite, and no studies were carried out on the fluorine release from WR and how any released fluorine at Ivittuut may affect the release and transport of other metals (Johansen et al. 1995, 2010).

Similar processes of sulfide-mineral controlled metal release have been documented at other large coastal and marine mine waste deposits. In Portman Bay (SE Spain), where sulfide-rich tailings were discarded directly into the marine environment, Martínez-Sánchez et al. (2008) showed that the mobility of Zn, Pb, and As is governed by the mineralogical composition of the sediments, including sulfides, jarosite, magnetite, and silicate phases. Sequential and selective extractions demonstrated that metals can be effectively immobilized in stable secondary phases such as jarosites, even under acidic conditions, whereas sulfide-bearing fractions represent a latent risk because oxidation can generate sulfuric acid and mobilize associated metals if sediments are disturbed or exposed to oxygen.

Laboratory leaching studies further indicate that Pb, Zn, and Cd mobility from contaminated sediments and waste materials depends not only on sulfide oxidation but also on coupled geochemical processes. Using long-term pH-controlled kinetic experiments, Payán et al. (2013) and Martín-Torre et al. (2016, 2017) showed that Fe availability, ionic competition, and pH strongly control the Pb and Cd release from Zn-rich materials, causing delayed or suppressed metal release at near-neutral pH. Their kinetic modeling indicates that reactions involving Fe and Zn regulate trace-metal release rates under saline conditions.

Thermodynamic and field-based studies also indicate that the stability of Pb- and Zn-bearing minerals is sensitive to physicochemical conditions. Using PHREEQC-based solubility modeling constrained by freshwater stream chemistry from several mining districts, Miler et al. (2022) showed that Pb- and Zn-bearing phases, including galena, sphalerite, cerussite, smithsonite, and sulfate minerals, exhibit markedly different stability fields as a function of pH, redox conditions, lithology, and temperature. In carbonate-rich systems, sphalerite and galena are relatively stable while carbonate and sulfate phases dominate metal release, whereas in silicate-rich environments cerussite and sphalerite become more soluble and contribute more strongly to Pb and Zn mobility. Their results further indicate that temperature variations can shift mineral solubility equilibria, increasing the solubility of phases such as sphalerite and cerussite and thereby enhancing metal mobility.

These studies show that Pb and Zn mobility is commonly controlled by sulfide-related and secondary mineral phases, even when galena and sphalerite occur only in very small amounts. Pb and Zn can therefore remain mobile in coastal and marine environments, but their release is governed by coupled geochemical and mineralogical controls rather than by sulfide oxidation only. However, the combined influence of seawater chemistry and low Arctic temperatures on these processes remains poorly constrained, particularly for cryolite-, galena-, and sphalerite-bearing WR such as that present at Ivittuut. In the present study, we apply a laboratory-based 40-week leaching experiment to study the following:

- The influence of temperature on the mineral weathering of the sulfidic waste rock at Ivittuut under seawater leaching conditions,
- Metals and fluoride behavior and leachability of the waste rock from Ivittuut.

## Materials and methods

### Study area

**Fig. 1:**
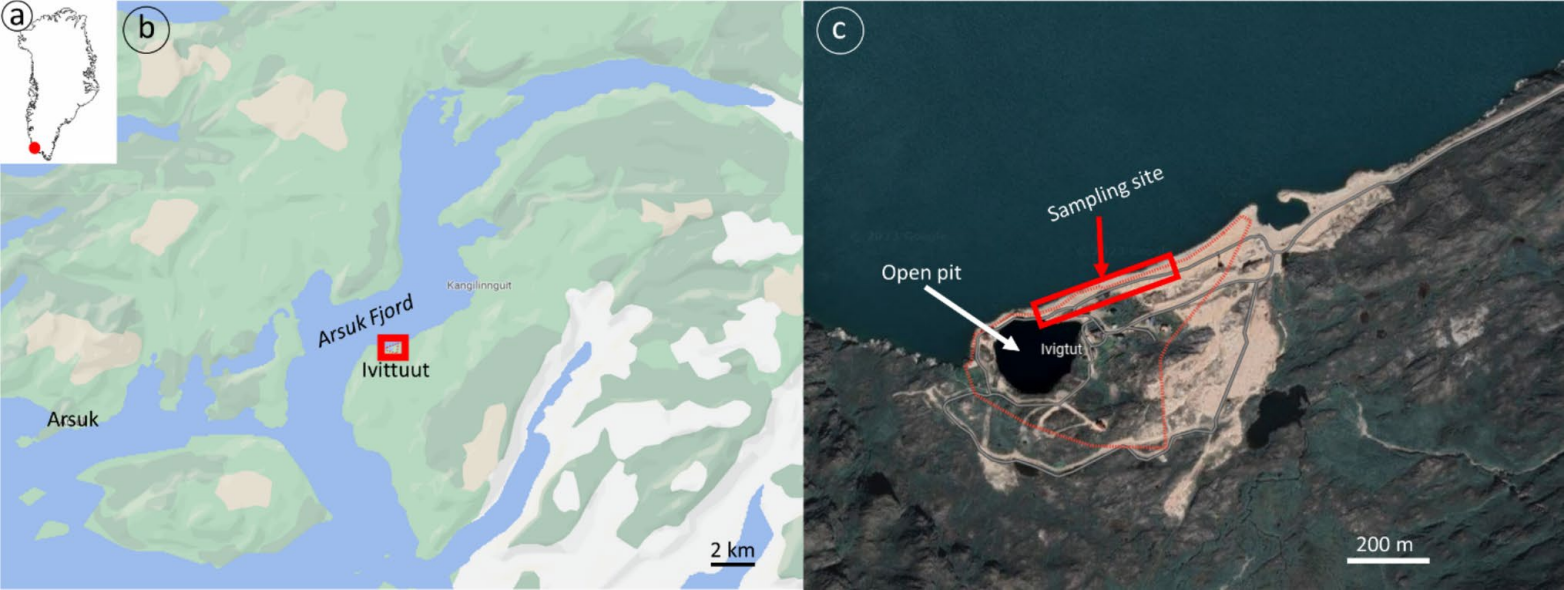
Map of Ivittuut: **a** overview map of Greenland, **b** map of Arsuk Fjord, and **c** map of Ivittuut mine site, annotated with sampling site and the open pit

Ivittuut is an abandoned mine site located in South Greenland (Fig. 1). The Ivittuut deposit is a unique hydrothermally developed cryolite body and belongs to the Gardar Province (Karup-Møller and Pauly 1979; Köhler et al. 2008). The open pit from which cryolite was extracted has dimensions of 115 × 50 m and reaches a depth of 70 m (Johansen et al. 1995). Cryolite was mined from 1854 to 1987 through open-pit mining (Fig. 1c) and used for aluminum production (Johansen et al. 1995, 2010; Larsen and Geertz-Hansen 2016). The cryolite ore was blasted, crushed, and sorted on-site and transported to Denmark for processing (Johansen et al. 2010; Søndergaard and Mosbech 2022). Extraction of waste rock was necessary to access the high-purity cryolite. The waste rock at Ivittuut consists of granite and greisen as well as low-grade ore, containing minerals such as cryolite, Pb, Zn, Cu, and Fe sulfides (galena, sphalerite, pyrite, and chalcopyrite), siderite (FeCO_3_), and quartz (SiO_2_). Some of the waste rock was used for infrastructure development at the mine site, mostly as a fill to construct roads, the quay, a constructive barrier between the open pit and the Arsuk Fjord, and disposed of along the coast of Arsuk Fjord (Johansen et al. 1995).

At Ivittuut, the coldest month of the year is January, with an average temperature of −11.1 °C at night and −4.4 °C during the day. The warmest month is July, with an average temperature of 5.6 °C at night and 13.9 °C during the day (Weather Atlas 2022).

### Sampling and preparation of waste rock

Waste rock (WR) samples were collected in September 2017 at Ivittuut from the quay area within the terrestrial environment at the surface (0–10 cm depth, Fig. 1c). The WR samples were transported to the Greenland Institute of Natural Resources (GINR) and air-dried prior to performing the experiments. The WR was crushed using a Retsch jaw crusher (Pulverisette 1, Model II) and sieved with a mesh size of 4 mm.

### Leaching solution

Seawater (SW) was used as a leaching solution and was collected every 2 weeks from the same position in the Nuuk Fjord (64°07′N, 51°53′W) at 50 m depth using a Niskin water sampler. The 50-m depth was chosen to avoid the influence of freshwater due to the snow melt. The SW was filtered using a 1.2-µm Whatman glass microfiber filter (grade GF/C) and was stored at 5 °C until use.

### Humidity cell test

#### Experimental setup

**Fig. 2:**
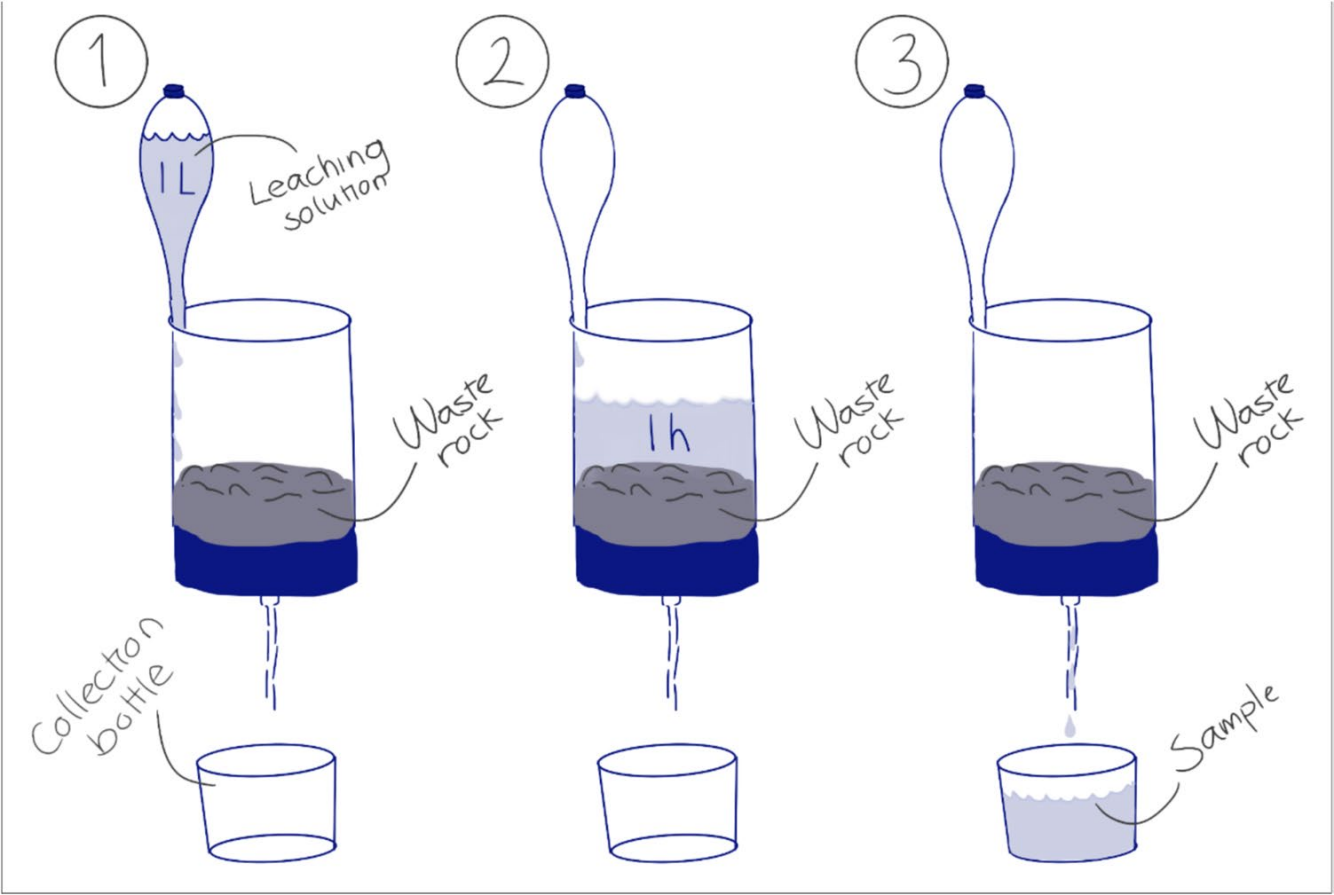
Experimental setup: (**1**) Flushing with leaching solution, (**2**) Leaching solution-WR contact for 1 h, and (**3**) draining leachate sample into a collection bottle

The humidity cell tests (HCT) consist of a column filled with WR through which water percolates (Fig. 2). The water was collected at the bottom after it passed the column and had been in contact with the WR for 1 h. The experiment was performed at the GINR using five plastic cells, according to the standard procedure D5744-13, option B, with modifications to accommodate different temperatures and the leaching solution (ASTM 2013).

The five HCT cells include the following:

- HCT0, a SW blank performed at room temperature without WR in the cell.
- HCT1 and HCT2 carried out at room temperature.
- HCT3 and HCT4 carried out in a climate chamber at 2 °C.

The plastic columns for the experiment have an inner diameter of 10.3 cm and a height of 20.3 cm. The bottom of the column had a 3-cm-thick filter sponge topped with a layer of nylon mesh with a pore size of 0.44 µm. They were rinsed with 2% nitric acid (HNO_3_) and deionized water before they were filled with 1000 g crushed and homogenized WR (≤ 4 mm) that was mixed thoroughly to avoid grain size and mineralogical stratification. A 1000 mL of SW was passed through the columns each week for 40 weeks. During each flush, the separatory funnel was opened, allowing the solution to flow steadily into the cells to avoid WR agitation. The solution remained in contact with the WR for 1 h in the cells. The cells were then drained overnight. The next day, the drainage tubes were closed, and cells opened, and the leachates and cells were weighed. Then the cells were left open to air-dry before 1000 mL SW was added again the following week.

The weekly collected leachates were filtered with a 0.45-µm nylon filter prior to chemical and physicochemical analysis.

The weight of WR, leaching solution volume, and temperature for each cell are shown in Table 1.

**Table 1:** Overview of HCT cell numbers, WR weight, type of leaching solution, experimental temperature, experiment length, and start and end date

Cell No.	Waste rock (g)	Leaching solution	Volume of leaching solution added every cycle (mL)	Temperature	Experiment length	Start date	End date
HCT0	0	Seawater	1000	23.5 °C	40 weeks	21/03/18	09/01/19
HCT1 HCT2	1000	Seawater	1000	23.5 °C	40 weeks	21/03/18	09/01/19
HCT3 HCT4	1000	Seawater	1000	2 °C	40 weeks	21/03/18	09/01/19

The room temperature and 2 °C leaching experiments were conducted identically except for the temperature difference. The room temperature had an average temperature of 23.5 ± 1.7 °C and humidity of 21 ± 6.4%, while the 2 °C treatment had an average temperature of 1.5 ± 0.6 °C and humidity of 53.9 ± 15.9%.

#### Analysis of physiochemical parameters

The pH, temperature, dissolved oxygen (DO), electrical conductivity (EC), and oxidation–reduction potential (ORP) were measured in each leachate. The temperature, pH, and ORP were measured using a Multi 9620 Intrusion Detection System multimeter (Multi 9620 IDS) equipped with a Yellow Springs Instruments (YSI) 4210 (for ORP measurement) and YSI 4110 probe (for pH and temperature measurements). The DO and EC were measured using a YSI Professional Plus (Pro Plus) Multi-parameter Instrument equipped with a YSI 5560 and a DO pro 2002 15J100535 probe. The pH probe was calibrated prior to the measurements and was tested in buffer solutions before each measurement. If the buffer solution measurements deviated by >  ± 0.2, the probe was re-calibrated. The EC probe was calibrated at the beginning of the leachate measurements and several times during the measurement of all leachates.

#### Chemical analysis of leachates

Approximately 30 mL of each leachate and blank was sampled from weeks 0–1–2–6–11–16–17–21–22–26–27–31–33–39–40 to be analyzed with Inductively Coupled Plasma Mass Spectrometry (ICP-MS). The ICP-MS analysis was performed at Eurofins Miljø A/S, Vejen, Denmark, using the standard protocol DS/EN ISO 17294 m:2005 ICP-MS.

The ICP-MS analysis included the measurements of the following elements: As, Ba, Pb, Br, Cr, Li, Ni, V, Sn, Co, Mo, Ag, Sb, Cd, Hg, Rb, Sr, Ga, Se, B, Al, Fe, Cu, Mn, Zn, I, Be, Y, Zr, Nb, Ru, Rh, Pd, Te, Ce, Pr, Nd, Sm, Eu, Gd, Tb, Dy, Ho, Er, Tm, Yb, Lu, Hf, Ta, W, Re, Os, Pt, Tl, Bi, Th, U, and Cs. Additionally, anions and cations such as Cl^−^, F^−^, SO_4_^2−^, Fe, Mg, Mn, and Na were measured in the leachate of week 40.

Free ionic F measurements were done using the Orion Dual Star pH/ISE meter with electrode stand (Cat. No. 2115000) equipped with a Fluoride combination electrode (Cat. No. 9609BNWP). Leachates were subsampled and each 50-mL subsample was mixed with 5-mL TISAB III ionic strength adjustor and measured with the probe.

Pb, Zn, and F were considered the primary target elements of this study, whereas other trace metals (e.g., Cd, Cu, As, Cr, Ni, Co, and Hg) were reported to provide additional context regarding potential environmental risk.

#### Data processing

**Fig. 3:**
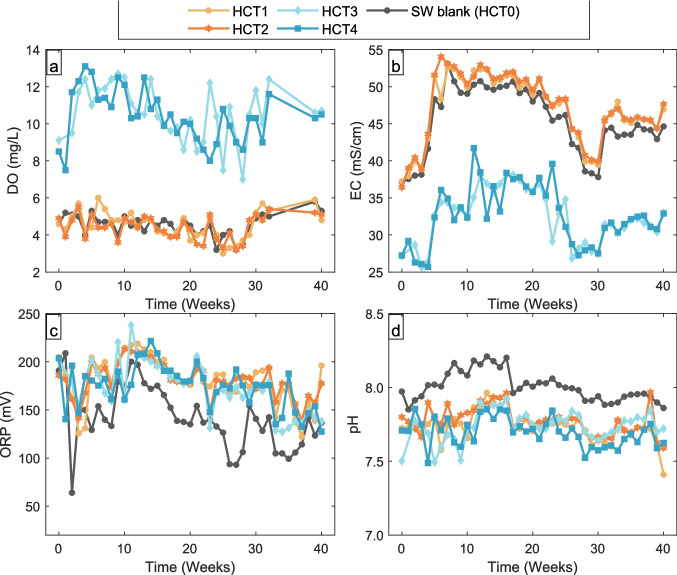
**a** Dissolved oxygen (DO) in mg/L, **b** electric conductivity (EC) in mS/cm, **c** oxidation reduction potential (ORP) in millivolt (mV), and **d** pH of HCT0-HCT4 through week 0 to 40. In the figure, HCT0 shows the experimental results for SW blank, while HCT1 and HCT2 and HCT3 and HCT4 show the results performed at 23.5 °C and 2 °C, respectively

The median concentration of each element in the seawater blank (Hum0) was subtracted from the corresponding values in all leachates (nHCT). In Fig. 3, concentrations of elements below the detection limit (DL) are represented by the DL value.

## Results

### Characterization of the leaching solution

The average ± standard deviation (SD) of physicochemical parameters and elemental concentrations of the SW blanks are shown in Tables 2 and 3. In the SW blank, all of the measured elements except Ba, Pb, Zn, Br, Li, As, Mo, Rb, Sr, Ga, B, I, and Cs are below the DL (Table 3, Supplementary material B). In the SW blank, concentrations of Pb, Zn and Cd exceed the GWQC for SW, whereas concentrations of F, Cu, As, Cr, Ni, and Co remain below the respective criteria (see Table 3).

**Table 2:** Average ± SD values of physicochemical parameters of the leaching solution (SW)

	Unit	SW (*n* = 40)
pH		7.8 ± 0.1
ORP	*(mV)*	159.0 ± 36.0
Electric conductivity	*(mS/cm)*	47.5 ± 3.9
DO	*(mg/L)*	7.5 ± 2.9

**Table 3:** Concentrations of selected chemical elements in SW blanks and Greenlandic Water Quality Concentrations (GWQC) guideline values (MRA 2015)

	SW blank	DL	GWQC in SW
	*(n* = *15, µg/L)*	*(µg/L)*	*(µg/L)*
Pb	2.23 ± 0.73	0.2	2
Zn	36.05 ± 17.21	5	10
F	630 ± 21.61	500	1500
Cu	< 3	3	2
As	1.93 ± 0.57	1	5
Cr	2.87 ± 2.50	1	3
Ni	2.35 ± 0.57	1	5
Co	< 1	1	-
Cd	2.00 ± 1.69	0.1	0.2
Hg	< 0.1	0.1	0.05

### Leachate characterization

#### Physicochemical parameters

**Fig. 4:**
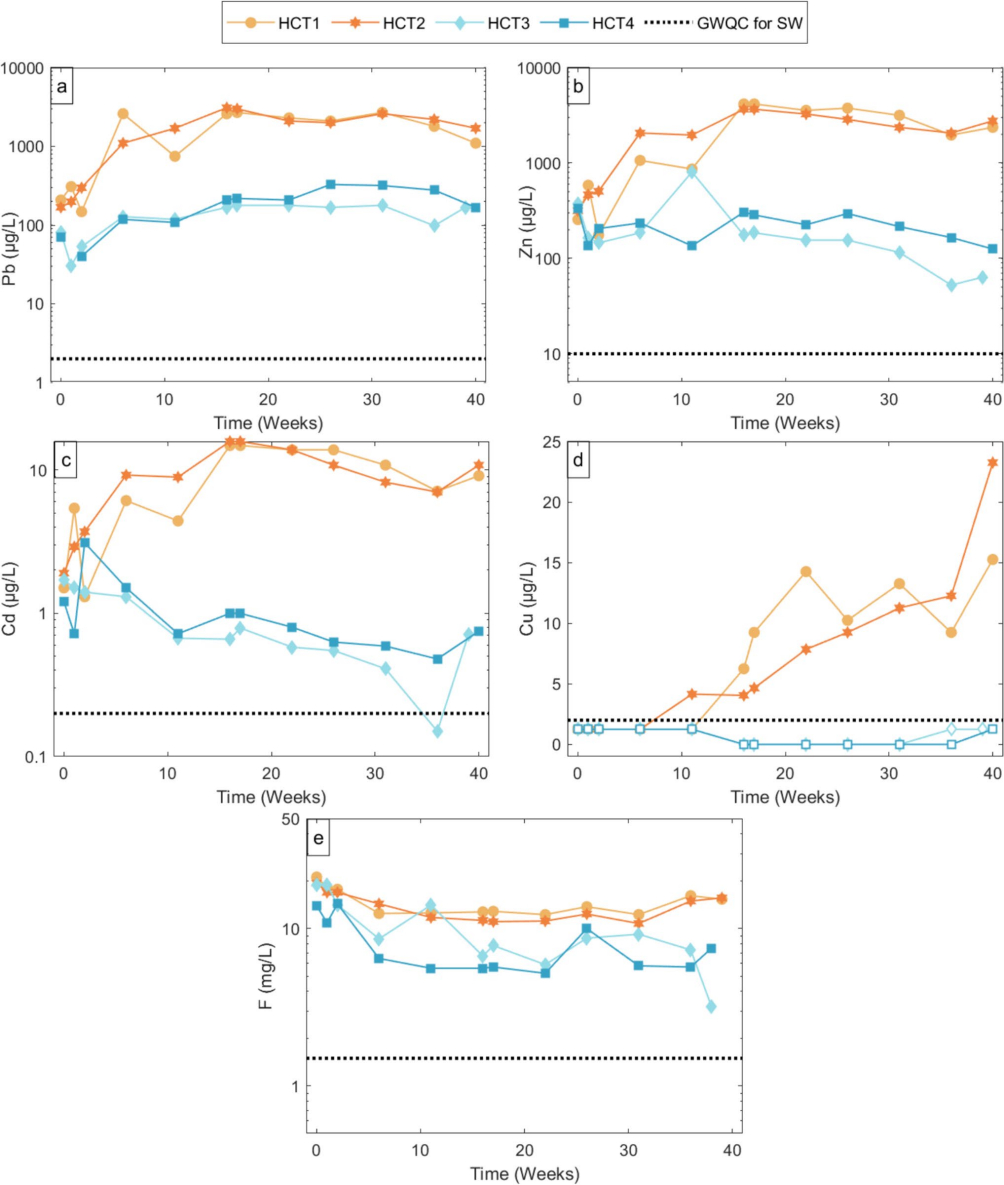
Log scale of Pb, Zn, Cd, Ni, Cu, and F concentrations in leachates of HCT1, HCT2, HCT3 and HCT4. The hollow markers on the figure indicate concentration levels approaching DL. In the figure, HCT1 and HCT2, and HCT3 and HCT4 show experimental results performed at 23.5 °C and 2 °C, respectively. The dotted black line shows the GWQC in SW for each individual element

Physicochemical parameters in all HCT leachates were measured every week. The elemental concentrations in the selected weeks and the physicochemical parameters are given in Figs. 3 and 4.

Results for the room temperature treatment (HCT1 and HCT2): The DO in the leachates from HCT1 and HCT2 remain stable throughout the 40-week period, with values averaging 4.5 ± 0.7 mg/L. These values are the same as values measured for the SW blank (DO of 4.6 ± 0.6 mg/L, Fig. 3a, Table 2). The EC values for HCT1 and HCT2 leachates are slightly higher compared to the SW blank (HCT0) by approximately 1 mS/cm, with an average value of approx. 50 mS/cm (Fig. 3b). The EC increases from week 0 to week 6, stabilizing approx. at 50 mS/cm, and declines in week 21, reaching approx. 45 mS/cm towards the end of the experiment (Fig. 3b).

The ORP in leachates HCT1 and HCT2 ranges from 150 to 200 mV, being consistent throughout the experiment (Fig. 3c, Table 2). The ORP of the HCT0 leachate varies between 100 and 200 mV (Fig. 3c). The pH of the HCT1 and HCT2 leachates varies between 7.4 and 7.9, showing minimal fluctuation over the duration of the experiment. This is slightly lower than the pH of the SW blank, which varies between 7.8 and 8.2 (Fig. 3d).

Results for the 2 °C treatment (HCT3 and HCT4): In contrast to HCT1 and HCT2, the leachates from the 2 °C treatment (HCT3 and HCT4) show a significantly higher DO value, averaging approx. 10.5 ± 1.4 mg/L (Fig. 3a). The overall trend of the DO values is like that of the SW blank, although it fluctuates (Fig. 3a, Table 2). The DO in leachates starts relatively low (approx. 8.8 ± 0.3 mg/L) and increases (approx. 12.0 ± 0.3 mg/L) at week 3, thereafter slowly decreasing (approx. 9.6 ± 0.7 mg/L) until week 24 when it increases (10.6 ± 0.1 mg/L) again towards week 40 (Fig. 3a). The EC values of HCT3 and HCT4 leachates are approx. 10 mS/cm lower than the SW blank (HCT0) and the HCT1 and HCT2 (Fig. 3b, Table 2).

The ORP values in HCT3 and HCT4 range from 150 to 200 mV and follow the trend observed in the room temperature treatment (Fig. 3c). The pH of HCT3 and HCT4 leachates is consistent with that of the room temperature leachates, ranging from 7.4 to 7.9 (Fig. 3d).

#### Leachate chemical composition

The absolute leached element concentrations were calculated by subtracting the median concentration of each element in the seawater blank (Hum0) from the measured concentrations of the leachates. Some elements have concentrations below the DLs (Supplementary material A). To show these in the figures, the value of the DL is provided as hollow markers (Fig. 4).

Results for the room temperature treatment (HCT1 and HCT2): Concentrations of Pb, Zn, and Cd in the leachates generated at room temperature were approx. 10 times higher than those in leachates generated in the 2 °C treatment (Fig. 4). For the other elements analyzed, e.g., Ni, Cr, and Co, the concentration differences in leachates at room temperature vs 2 °C are minor (Supplementary material A). Cobalt concentrations are only detected in the first 2 weeks and then drop below the concentrations measured in the SW blank (lower than 0.43 µg/L, Supplementary material A). Chromium is detected after week 16, varying between 1 and 3 µg/L (Supplementary material A). The Ni concentrations fluctuate close to the values for the blank (approx. 1.38 µg/L) throughout the experimental period, with no clear leaching pattern (Supplementary material A). The concentration of As and Hg in the SW blank are higher than in the leachates (Supplementary material A).

The Pb, Zn, and Cd concentrations in the leachates from the room temperature treatment show a gradual increase during the initial weeks of the experiment, reaching maximum levels at week, corresponding to an approximately order-of-magnitude increase relative to early-stage concentrations. After reaching these maxima, the concentrations slowly decrease towards week 36, although Zn and Cd show a secondary increase toward the end of the experiment (Fig. 4, Supplementary material A). The fluorine (F) leaching pattern is the opposite to that of Pb, Zn, and Cd. The F concentrations in leachates are elevated at the start of the experiment, followed by a decrease until week 17 and a slight increase towards week 39 (Fig. 4e, Supplementary material A).

The Cu concentrations in the leachates were below the detection limit of 3 µg/L during the initial weeks (0 to 4) and first exceeded the detection limit at weeks 6 and 11 for the two cells at room temperature. Concentrations then increased towards week 40, reaching maximum values of approximately 15 to 20 µg/L. Results for the 2 °C treatment (HCT3 and HCT4):

- The Pb concentrations increase during the first part of the experiment, reaching maximum levels around week 26, while Zn concentrations remain stable except for a single high value at week 11 (Fig. 4a, b, Supplementary material A).
- After week 26, both elements show decreasing trends toward the end of the experiment, with Zn concentrations falling below the initial levels, while Pb concentrations remain elevated relative to the start of the experiment (Fig. 4a, b, Supplementary material A).
- The Cd concentrations show slightly decreasing trends from 1 µg/L at the start of the experiment to reach at approx. 0.15–0.7 µg/L at the end of the experiment.
- The F concentrations are typically lower than those from room temperature treatment, except for the measurements at weeks 1 and 11, where at room temperature F concentrations exceed the values by 1–2 mg/L (Fig. 4e). The F concentrations in leachates are elevated at the start of the experiment and decreased over time until week 17, slightly decreasing towards the end of the experiment (Fig. 4e, Supplementary material A).
- The Cu concentrations remain below the DL (3 ug/L) throughout the 40-week experimental period.

## Discussion

### Temperature effect on leaching behavior

#### Physicochemical parameters

The DO, EC, ORP, and pH of the leachates at room temperature and 2 °C show trends like those in the SW blank (Table 2, Figs. 3 and 4). This suggests that the SW properties, such as high ionic strength and buffering capacity, have remained unchanged during interaction with the WR and control the physicochemical conditions. A similar observation was made by Texeira et al. (2023) in their study of the impact of seawater on mine wastes. The differences in DO and EC, however, are significant between the different temperature treatments. Dissolved oxygen levels in the 2 °C leachates are consistently higher than those at room temperature, which is expected because colder water dissolves more oxygen compared to warmer water (Addy and Green 1997). Electrical conductivity in 2 °C leachates is consistently lower than the ones measured in room temperature leachates and the SW blank, which is consistent with a lower leaching rate of salts under lower temperature. Kumar et al. (2023) reported that increased water temperature led to increased conductivity. The DO and EC values at room temperature are closer to those of the SW blank (also measured at room temperature), reinforcing the view that temperature has a direct impact on the DO.

The ORP and pH values in the room temperature and 2 °C treatment were within the experimental error, indicating that temperature had only a minor influence on these parameters (Fig. 3). Water-rock interactions increase ORP by approximately 50 mV and decrease pH by ~0.3 units in all treatments (Table 2, Fig. 3). ORP and pH are closely related, as lower pH values correspond to higher ORP due to the presence of free H⁺ ions in solution (Chang et al. 2002; Van Haute et al. 2019). The pH decrease in the leachates contributes to the observed ORP increase. This pH reduction results from the leaching of various mineral phases in the WR. Specifically, the dissolution of siderite in oxidizing environments decreases pH due to carbonic acid formation (Morin and Cherry 1986; Skousen et al. 1997).

#### Cation and anion leaching

Temperature is found to have an important impact on the leaching rate of Pb, Zn, and F from the WR (Fig. 4). At room temperature, Pb, Zn, and Cd concentrations increased 15-, 14-, and 9-fold, respectively, from the initial leachate to their peak values, whereas in the 2 °C treatment the increases were only 3-, 1-, and 0.4-fold, respectively. The peak concentrations at room temperature were 2848 ± 212 µg/L, 3916 ± 354 µg/L, and 15.3 ± 0.7 µg/L, respectively, while at 2 °C, Pb peaked at week 27 (248 ± 113 µg/L). These ratios indicate that elevated temperatures accelerate metal release compared to colder conditions (Fig. 4). This behavior is consistent with increased dissolution kinetics and solubility of metal-bearing minerals at elevated temperatures, which is well documented in geochemical studies (Barrett and Anderson 1988; Dutrizac 1992; Peters 1992). Barrett and Anderson (1988) found that the solubility of galena and sphalerite in NaCl brines increased with increasing temperatures. They highlighted that an increase from 25 to 60 °C resulted in an increase in the solubility of minerals.

In the room temperature treatment, the concentrations of Pb, Zn, and Cd peak at around week 17 and decrease slightly afterwards. The small decrease in metal concentrations in the leachates is interpreted as a change in the reactivity between the mineral surfaces and the SW. The formation of secondary minerals coating galena crystal surfaces has been observed in natural samples from Ivittuut (Baba and Adekola 2011; Dutrizac and Chen 1990). If the formation of secondary minerals occurred during the time of our experiments, this could explain the decreased release after week 17. Dutrizac and Chen (1990) described PbCl_2_ (cotunnite) forming on the surface of galena, preventing further dissolution during leaching in a ferric chloride media.

Leachates from the 2 °C treatment show overall lower concentrations of Pb, Zn, and Cd compared to leachates generated at room temperature. Equally, Pb concentrations, which peak at week 26, are approx. 11 times lower than that of the room temperature, which peaked at week 17 (248.1 ± 113.1 µg/L). This is followed by a decline towards the end of the experiment, while Zn and Cd show no distinct peaks and steadily decline from the start of the experiment. This behavior suggests that lower temperatures reduce the leaching rates of Pb, Zn, and Cd and hence immobilized metals, likely due to the low dissolution rates of minerals at low temperatures (Fu et al. 2023; Lu et al. 2021). The coupled decrease of Zn and Cd further suggests a mineralogical control, as both elements are hosted in sphalerite, whose dissolution appears to be more strongly suppressed at 2 °C than at room temperature, unlike other contributing mineral phases. The decreased leaching rate at 2 °C highlights temperature as a key control on metal release from mining waste, particularly under Arctic conditions and during periods of seasonal cooling. Fluorine concentrations are twice as high at room temperature than in the 2 °C treatment, but the overall pattern is the opposite to Pb and Zn. Fluorine concentrations are highest in the leachates from the first week and decrease thereafter until week 17 for both room temperature and 2 °C treatment. After week 17, the F concentrations stabilize for each treatment. Nevertheless, the absolute change in F concentration is not large and remains relatively constant at 12.8 ± 0.2 mg/L over the 40 weeks. The lower F concentrations at 2 °C suggest that temperature also influences F solubility, although the F leaching process differs from cations, such as Pb and Zn. The initial high F release is consistent with rapid dissolution cryolite grains, which dominates early in the leaching process. The subsequent decrease reflects the exhaustion of the readily accessible surfaces of these grains, and surface-controlled dissolution therefore explains the observed F leaching pattern. As an ionic solid, cryolite is readily crushed to a fine powder, potentially contributing to the pool of readily soluble material in the WR in the column.

There is limited research on the solubility of metals and, in particular, cryolite in seawater. Existing studies on cryolite solubility have been conducted only in freshwater, with an estimated solubility product constant (Log(Ksp)) of −34.0 ± 0.3 (Nordstrom 2022; Roberson and Hem 1973). This indicates that cryolite is highly insoluble in freshwater. However, the fluoride concentrations in all leachates from this experiment suggest significant dissolution of a fluoride mineral. Moreover, cryolite is observed to be strongly weathered in WR compared to other minerals at the Ivittuut site (Jeremiassen et al., in prep.). 

The solubility of galena and sphalerite has been studied in more detail than that of cryolite. Barrett and Anderson (1988) found that higher NaCl molality in a leaching solution increases the solubility product constant for both. However, Barrett and Anderson (1988) examined NaCl with molalities of 1–5 M, much higher than that of natural seawater, which is typically around 0.5 M (Von Damm et al. 1991).

The increasing Cu concentration observed in the room temperature leachates suggests that the Cu in chalcopyrite is more susceptible to leaching at room temperature than at 2 °C, at which the Cu concentrations remained below the DL throughout the 40-week period.

The relatively stable leachate concentrations of Ni, Cr, and Co across both temperature conditions suggest that these elements are not water-soluble in the minerals in the WR and their leachability is not substantially affected by the presence of competing ions or temperature variations.

The concentrations of As and Hg in the leachates are lower than in the SW blank. This indicates that these elements are retained in the column and are adsorbed on mineral surfaces.

### Effect of seawater chemistry on the leaching process

The use of SW instead of distilled water as the leaching solution mimics the natural environmental weathering process of the Ivittuut WR in contact with the Arsuk Fjord water, but introduces additional complexities due to the presence of high salt content and competing ions such as Cl^−^, SO_4_^2−^, and Mg^2+^ (Azevedo Schueler et al. 2023). Additionally, using the same SW throughout the experiment would have been ideal, but this was not feasible due to the volume required. As a result, SW was subsequently sampled, leading to variations in the composition of SW over time. Various ions (Cl^−^, SO_4_^2−^, CO_3_^2−^, and OH^−^, etc.) can form complexes with metals with different complexation affinities (Millero et al. 2009). Lead and zinc form complexes with Cl ions in seawater, and complexation dictates metal speciation, which affects the solubility of metals and hence the leaching rates (Bruland 1989; Millero et al. 2009; Texeira et al. 2023). Therefore, the Cl-complexes in seawater enhance the solubility of metals, increasing the metal concentrations in leachates.

At warm temperatures, Pb forms more stable chloride complexes in a solution, as the stability constant for these complexes increases with temperature (Luo and Millero 2007; Seward 1984). Luo and Millero (2007) showed increasing stability constants for Pb-Cl complexation with increasing temperature between 15 and 45 °C. In contrast, at lower temperatures, the solubility of metal-bearing minerals, such as zinc sulfide (sphalerite) or lead sulfide (galena), decreased, resulting in a reduced release of Pb and Zn from minerals into the leachate (Barrett and Anderson 1988; Dutrizac 1992; Mahyar et al. 2018; Peters 1992). Additionally, colder temperatures can alter the ionic strength and saturation state of the solution, shifting the equilibrium towards the precipitation of secondary minerals, particularly when the system reaches saturation for specific metals (He et al. 2009).

### Environmental implications and future recommendations

The risk of metal mobilization may be significantly lower in cold Arctic conditions compared to mid-latitude or tropical regions due to the temperature-dependent release of metals from minerals such as galena and sphalerite in mine waste. This has important implications for the environmental risk assessment at Ivittuut, as temperature fluctuations will cause seasonal shifts in metal leaching rates. These findings may apply at other mine sites, but other factors such as ore mineralogy, waste management, and site-specific parameters will also play a role in the leachability, mobility, and dispersion of these elements into the environment. While Pb, Zn, and F represent the dominant contaminants of concern at Ivittuut based on historical monitoring, other elements provide additional insights into trace-element behavior under Arctic weathering conditions.

Although absolute concentrations were low, elements such as and Hg remained below seawater blank levels throughout the experiment, suggesting effective retention within the WR columns, like through adsorption onto secondary mineral surfaces or incorporation into newly formed phases. Although these elements do not appear to pose an immediate leaching risk under the tested conditions, their immobilization may be sensitive to future changes in geochemical conditions, such as shifts in redox potential, pH, or salinity associated with climate-driven changes in hydrology or increased weathering.

Other trace metals, including Ni, Cr, and Co, showed concentrations close to the seawater blank values, indicating limited mobilization under the experimental conditions. Their low release may reflect the low content of the elements in the minerals tested.

The halting or reduction in Pb, Zn and F release at freezing or near-freezing temperatures suggests that Arctic climatic conditions slow down the metal mobilization, reducing the risk of Pb and Zn release at Ivittuut during winter months. Nevertheless, during the past few winters due to climate change, there were warm weather spells, which led to temperatures above freezing, resulting in continued leaching processes during the winter months as well. The snowmelt in the spring causes large flushes in a short time span but would simultaneously dilute the metal concentrations with higher volumes of freshwater (Jia et al. 2023). During the summertime the average temperatures are 5.6 °C at night and 13.9 °C during the day. Leaching rates are predicted to fall between the ones we observe for the room temperature and the 2 °C treatments.

It is important to note that HCT experiments use fixed weekly seawater additions with defined water-WR contact times and drying intervals. In the field, seawater infiltration into the Ivittuut WR may occur more frequently than once per week, implying that leaching rates in the experiments could be underestimated in the experiment. However, the WR in the experiment uses much smaller grain size than the field material. This increases the surface area and promotes faster dissolution, meaning that while the experiment may underestimate the leaching capacity, it might simultaneously overestimate the absolute leaching of elements compared to field conditions at the Ivittuut site. These factors indicate that the HCT results provide a useful indication of relative trends in leaching and temperature effects but should be interpreted with caution when estimating absolute release under the field conditions.

Site hydrology also plays an important role in controlling leaching rates and the release of elements into the environment. In the field, the coarse waste rock is highly infiltratable, allowing seawater to penetrate, while inland areas may predominantly receive freshwater infiltration, and intermediate zones may contain brackish water (Larsen and Geertz-Hansen 2016). These natural hydrological conditions produce variable flow paths and residence times that differ from the fixed weekly water additions used in the HCT experiments.

Understanding the role of temperature and the interaction between seawater composition and mineralogy is essential for predicting long-term metal behavior in marine-influenced environments in relation to mine waste remediation and mine drainage mitigation. The present experimental results show that the contact of WR with seawater at Ivittuut accelerates mineral weathering in comparison to contact with freshwater (Jia et al. 2023). These results need to be considered when remediating or disposing sulfide-bearing waste rocks at mine sites.

## Conclusions

This study investigates the influence of temperature and seawater on the leaching behavior of metals and fluoride from the waste rock at Ivittuut, in South Greenland. The results show that temperature plays an important role in controlling the release of contaminants, with warmer temperatures promoting faster metal leaching and colder temperatures suppressing metal mobilization. Higher concentrations of Pb, Zn, and Cd are observed at room temperature, while lower concentrations are recorded at 2 °C. The release of the metals leads to elevated concentrations of Pb and Zn in the Arsuk Fjord, which significantly exceeds the GWQC threshold (MRA 2015). The temperature effect on F release also mirrors the trends seen with the metals, though its leaching pattern was distinct, suggesting different mechanisms. The highest fluoride concentration in leachates is 14 times higher than the GWQC threshold. Such levels of Pb, Zn, and F are considered harmful to biota.

Water-WR interactions at both temperatures caused slight shifts in pH and ORP with 0.3 and 50 mV, respectively, which we link to the dissolution of minerals such as siderite.

The use of seawater as a leaching solution reflects natural weathering processes at the coast of Ivittuut, as it contains higher salinity and competing ions, such as chloride and sulfate. These ions may enhance the metal solubility and influence leaching behavior.

Colder temperatures in Arctic and subarctic climates than in other regions act as a natural barrier to metal release, reducing the environmental risk of contamination during the winter months. However, ongoing climate change and seasonal temperature fluctuations may still enhance the leaching of contaminants into the environment. This emphasizes the need for more detailed investigations into the long-term behavior of mine waste, to develop proper mine waste remediation strategies and to enhance environmental risk assessments.

## Supplementary Information

Below is the link to the electronic supplementary material.

ESM 1 (CSV.3.50 KB)

ESM 2 (CSV.18.1 KB)

## Data Availability

The authors declare that the data supporting the findings of this study are available within the paper and its Supplementary Information files.
